# The Prevalence of Educational Neuromyths among Hungarian Pre-Service Teachers

**DOI:** 10.3390/jintelligence11020031

**Published:** 2023-02-03

**Authors:** Julianna Vig, László Révész, Mónika Kaj, Katalin Kälbli, Bernadett Svraka, Kinga Révész-Kiszela, Tamás Csányi

**Affiliations:** 1 Institute for the Psychology of Special Needs, Bárczi Gusztáv Faculty of Special Needs Education, ELTE, Eötvös Loránd University, H-1097 Budapest, Hungary; 2 Institute of Sports Science, Eszterházy Károly Catholic University, H-3300 Eger, Hungary; 3 Hungarian School Sport Federation, H-1063 Budapest, Hungary; 4 Institute for the Methodology of Special Needs Education and Rehabilitation, Bárczi Gusztáv Faculty of Special Needs Education, ELTE, Eötvös Loránd University, H-1097 Budapest, Hungary; 5 Department of Physical Education Theory and Methodology, Hungarian University of Sports Science, H-1123 Budapest, Hungary; 6 Department of Education, Faculty of Primary and Pre-School Education, ELTE, Eötvös Loránd University, H-1126 Budapest, Hungary; 7 Doctoral School of Mental Health Sciences, Semmelweis University, H-1089 Budapest, Hungary; 8 Institute of Special Education, Eszterházy Károly Catholic University, H-3300 Eger, Hungary; 9 Department of Physical Education, Faculty of Primary and Pre-School Education, ELTE, Eötvös Loránd University, H-1126 Budapest, Hungary

**Keywords:** brain knowledge, education, neuroliteracy, neuromyths, pre-service teachers

## Abstract

Teachers with poor neuroliteracy fail to distinguish scientific evidence from neuromyths (NM), which might lead to the implementation of pseudoscientific educational methods. The prevalence of NM and general knowledge about the brain (GKAB) among in-service and pre-service teachers has been assessed in multiple countries, but no such study has been performed in Hungary. The aims of this study were to (1) assess the neuroliteracy of pre-service teachers, (2) compare the results with those of previous studies and (3) analyze the factors influencing neuroliteracy. Our sample included 822 pre-service teachers from 12 Hungarian universities. We developed a survey including 10 NM and 13 GKAB statements, adapted from a widely used questionnaire. The average rate of incorrect answers to NM was 56.9%, whereas the average rate of correct answers to GKAB was 70.9%. Male gender and frequency of using Facebook as the primary information source about neuroscience were the only predictors of NM acceptance. In comparison with other studies, the Hungarian pre-service teachers had the second highest endorsement of NM. The most prevalent NM were linked to motor functions, which might be related to the widespread use and promotion of motor therapies in Hungary.

## 1. Introduction

The growing need for developing teaching methods based on neuroscience and the emergence of a new research field, neuroeducation on the wake of this need has been observed in many countries (OECD 2002). However, the transfer of up-to-date neuroscientific knowledge to the field of public education has been shown to be moderately successful (Macdonald et al. 2017). Previous studies showed that among teachers as well as pre-service teachers interested in neuroeducation, there is a tendency to oversimplify, misunderstand or misinterpret the available scientific evidence (even the adjective “brain-based”, widely used for methods acclaimed to be based on neuroscience reflects such oversimplification) (Grospietsch and Lins 2021; Howard-Jones 2014). Failure to distinguish scientific evidence from so-called neuromyths (NM) might lead to the implementation of educational methods that are not evidence-based (Dekker et al. 2012; Ferrero et al. 2016; Pasquinelli 2012). Examples of such methods include the classification of students into ’visual’, ’auditory’ and ’kinesthetic’ types (Coffield 2004; Newton and Salvi 2020) or into ’left-brain’ and ’right-brain’ learners (Lindell and Kidd 2011), and teaching them accordingly.

Previous studies showed that although the growing popularity of neuromyths is a global phenomenon, certain aspects are culture-specific (Deligiannidi and Howard-Jones 2015; Ferrero et al. 2016; van Dijk and Lane 2020). A number of studies identified factors associated with either an increased or a decreased susceptibility to neuromyths (usually referred to as risk factors and protective factors, respectively). Protective factors include sound educational background (Macdonald et al. 2017; Ruhaak and Cook 2018; Zhang et al. 2019), access to new scientific evidence (Macdonald et al. 2017), completing science, neuroscience or psychology courses (Dündar and Gündüz 2016; Düvel et al. 2017), whereas risk factors include the marketing of “brain-based” programs and the “appetite” for brain-related news (Pasquinelli 2013). Findings related to the role of age and gender have been inconsistent (Dündar and Gündüz 2016; Ferrero et al. 2016; Hermida et al. 2016; Karakus et al. 2015). Identifying and analysing risk factors and protective factors is of key importance as it could help educators develop methods to “immunize” their students against acceptance of neuromyths (Carter et al. 2020).

In the past decade, the prevalence of neuromyths has been assessed in some Western European countries (Dekker et al. 2012), Southern Europe (Deligiannidi and Howard-Jones 2015; Dündar and Gündüz 2016; Ferrero et al. 2016; Karakus et al. 2015; Papadatou-Pastou et al. 2017; Rato et al. 2013), North America (Blanchette Sarrasin et al. 2019; Ruhaak and Cook 2018; van Dijk and Lane 2020) South America (Gleichgerrcht et al. 2015), Asia (Pei et al. 2015) and Australia (Carter et al. 2020; Kim and Sankey 2018). To our knowledge, only one Polish study assessed in-service teachers in Eastern European countries (Chojak et al. 2021), but our study would be the first to assess neuromyths in this region among pre-service teachers.

A recent review of studies related to educational neuromyths showed that the studies were mostly carried out on samples of in-service teachers, and few studies assessed the neuroliteracy and the predictors and protectors against neuromyths among pre-service teachers (Torrijos-Muelas et al. 2021). To our knowledge, data from European pre-service teachers are only available from Germany (Düvel et al. 2017; Grospietsch and Mayer 2019), Slovenia (Škraban et al. 2018), Austria (Krammer et al. 2019, 2021) Turkey (Canbulat and Kiriktas 2017; Dündar and Gündüz 2016), England (McMahon et al. 2019), Switzerland (Tardif et al. 2015), Greece (Papadatou-Pastou et al. 2017) and Spain (Fuentes and Risso 2015).

Collecting data from students at higher education training institutes is crucial because many teachers who endorse neuromyths tend to use instructional methods based on these misconceptions in their practice (Lethaby and Harries 2016). University courses are considered appropriate starting points to fight against neuromyths (Ferreira and Rodríguez 2022; Menz et al. 2021b), although university students may still hold on to their beliefs after completing a psychology or neuroscience course (Im et al. 2018; Menz et al. 2021a).

To our knowledge, no study assessed the prevalence of neuromyths among Hungarian pre-service or in-service teachers. For this reason, the aims of this study were to (1) assess the prevalence and acceptance of the best-known educational neuromyths and general knowledge about the brain (GKAB) among Hungarian pre-service teachers, (2) compare the results with those of previous studies performed in other countries and (3) analyze the factors that influence neuroscience literacy.

## 2. Materials and Methods

### 2.1. Participants

Our target population were Hungarian pre-service teachers learning as prospective preschool-, primary-, or special need education teachers in the academic year of 2021–22. To enroll participants, we contacted one lecturer from each university where the above-mentioned training took place based on the official data of the Hungarian Education Authority (www.felvi.hu).

In total, 12 out of the 16 lecturers responded positively to our initiative. They disseminated the survey via mailing lists used for official communication with students in February and March 2022.

Completion of the survey was voluntary and anonymous, which was stated in the description of the project included in the survey.

### 2.2. Survey

We created an online survey in Hungarian (using Google Forms), which consisted of a short description of the project, followed by two sets of questions and 44 neuroliteracy statements. The first set included demographic questions about age, gender, degree, and residence. The second set included questions about previous education (including courses related to psychology and neuroeducation), questions about the current interest in neuroscience and neuroscience-based educational methods and questions about the sources used for being informed about the above-mentioned topics. The questions were followed by 44 statements, including 23 statements related to brain function and learning, translated and adapted from the study of Dekker et al. (2012), and 21 additional statements related to neurobiology of motor development and learning foreign languages.

On the basis of the present article’s research aims, we only analyzed the neuroliteracy responses to the 23 statements adapted from the Dekker et al. questionnaire. Of these, 10 statements are educational neuromyths and 13 are statements related to general knowledge about the brain.

Similarly to the study by Grospietsch and Mayer (2019), we followed the methodological recommendations of Macdonald et al. (2017) and replaced the three-choice answer format (Correct/Incorrect/I don’t know) used by Dekker et al. (2012) with a 4-point Likert scale, and the responders were required to specify how sure they were of their answer (4 = Strongly agree, 3 = Somewhat agree, 2 = Somewhat disagree, 1 = Strongly disagree). (For each statement, the answer ‘I don’t know’ was also an option.)

Statements were translated and culturally adapted from English to Hungarian by the members of the research group with relevant qualifications, content knowledge, and proficiency in English, following the ITC recommendations (International Test Commission 2017). Some adjustments were made to ensure clarity of the content and to maintain a balanced ratio of correct and incorrect statements. Finally, we digitalized all survey items, and provided a random order of the neuroliteracy statements.

We conducted a pilot study for analyzing the intelligibility of the statements. Ten students were interviewed after completing the survey about their understanding of each statement. Four experts from the research group were responsible for the interviews, who collected the data about possible misunderstandings or other issues. Each interview took 40–50 min on average. Based on the results of the interviews, the statements were finalized with some minor modifications in the online survey.

### 2.3. Data Processing

Data cleaning process was conducted before the statistical analysis to improve data quality. In total n = 906 participants completed the survey within the two-month period. Participants were excluded if: (1) they were not a student of the 12 participated university (n = 42), (2) they were not learning as a pre-service preschool, primary school, physical education or special needs education teacher or other teacher specialization (e.g., language teacher) (n = 34), (3) any items of the survey were missing (n = 5), (4) any systematic error (e.g., all answers were the same) were explored through the data screening process (n = 3).

After the data cleaning process, responses for the 23 neuroliteracy statements were coded into a dummy format (agree/disagree/don’t know) from a 4-point Likert scale for better comparability with previous studies following recommendation of Grospietsch and Mayer (2019).

Finally, all answers were coded based on the correctness of each item (Table 1).

### 2.4. Statistical Analysis

Descriptive statistics were calculated for each educational neuromyth and general statement about the brain statements (frequency of correct, incorrect and uncertain (‘I don’t know’) responses) among participants. Shapiro–Wilk test was used to examine the normality of the continuous variables. As none of the examined dependent variables showed normal distribution, nonparametric tests were used for further analysis. The associations between the outcome (the percentage of incorrect answers) and the predetermined independent variables (gender, age, previous degree, academic year, completed psychological courses, current specialization in university education, previous courses related to neuroeducation, interest in neuroscience of learning/behavior, frequency of using information sources about neuroscience and GKAB error score) were estimated with multivariate ordinal logistic regression. Results are presented as Odds Ratio (OR) with corresponding 95% CIs. Statistical analyses were performed using SPSS statistical software (IBM SPSS Statistics, version 25 for Windows; Chicago, IL, USA). For all quantitative analysis, a statistical threshold of α = 0.05 was used.

## 3. Results

### 3.1. Demographic Characteristics of the Study Participants

The final sample comprised n = 822 participants, including 87.7% women and 11.7% men (0.6% did not specify their gender) from 12 Hungarian teacher training universities. Mean age of participants was 29.66 ± 9.93 years. At the time of the survey, all participants were enrolled in a university teacher training program, and 34.7% already had a degree from previous studies. Half of the sample (49.7%) were in the 1st or 2nd year of the training. Most participants (40.5%) were enrolled in a special needs education teacher program, followed by a preschool teacher (17.5%), primary school teacher (14.4%) and physical education teacher programs (13.6%). In terms of participants’ residency, all seven main regions of Hungary are represented in the sample (21.7% were from the capital, Budapest).

Demographic characteristics of the sample are presented in Table 2.

### 3.2. Previous Studies and Interest in Neuroscience

One-third (34.7%) of the participants already had a degree, including 18.1% with a degree in education. More than 96% had completed at least one psychology-related course (the majority, 65.1% have completed 3 psychology-related courses). Only 13% of participants stated that they have ever learnt about neuroeducation, but the great majority (92%) stated that they were interested in the neuroscientific background of learning and learning/behavioral disorders (including 26.6% who expressed “very much” interest) (Table 3).

### 3.3. Frequency of Using Various Sources of Information

**Figure 1:**
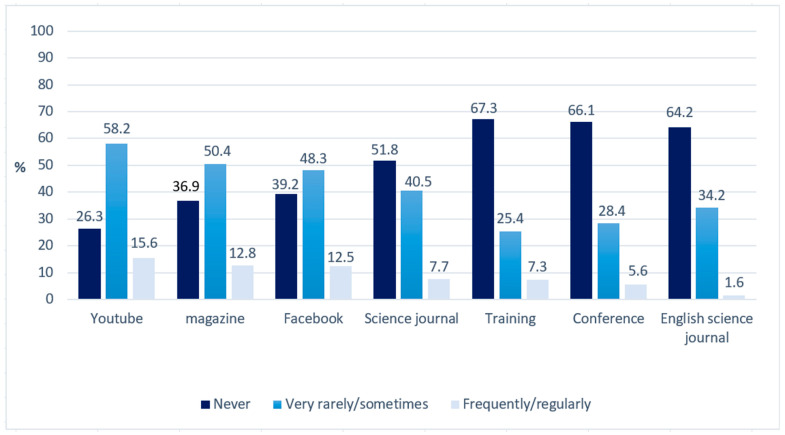
Frequency (%) of using various sources of information about neuroscience.

The most popular sources of information about neuroscience and education were YouTube and Hungarian-language magazines (73.8% and 63.2% stated that they used these at least sometimes, respectively), but even these sources were infrequently used by most participants. Only a small fraction of participants stated that they use any sources frequently or regularly (YouTube 15.6%, magazines 12.8%, Facebook 12.5%, scientific journals 7.7%, training 7.3%, conference 5.6%, English language scientific journals 1.6%) (Figure 1).

### 3.4. Prevalence and Ranking of Neuromyths

Table 4 shows the frequency of correct, incorrect and uncertain (‘I don’t know’) answers to each of the ten educational neuromyths, respectively. The data confirms that neuromyths about the brain are prevalent among Hungarian pre-service teachers. More than 60% of the participants falsely supported (that is, gave incorrect answers to) the following five neuromyths: “Exercises that rehearse co-ordination of motor-perception skills can improve literacy skills” (89.7%); “Children have learning styles that are dominated by particular senses.” (82.1%); “Short bouts of co-ordination exercises can improve integration of left and right hemispheric brain function.” (81.4%); “Children are less attentive after consuming sugary drinks and/or snacks.” (62.4%); “Differences in hemispheric dominance (left brain, right brain) can help explain individual differences amongst learners.” (60.0%).

The rates of uncertain answers to neuromyths varied between 7.2% and 18.2%. The highest rates of ‘I don’t know’ answers were given for the following three neuromyths: “We only use 10% of our brain” (18.2%); “Differences in hemispheric dominance (left brain, right brain) can help explain individual differences amongst learners.” (15.2%); “Short bouts of co-ordination exercises can improve integration of left and right hemispheric brain function.” (15.1%). The neuromyth about which the least participants were uncertain was the following: “Children must acquire their native language before a second language is learned. If they do not do so, neither language will be fully acquired.” (7.2%).

### 3.5. Prevalence and Ranking of Statements Related to General Knowledge about the Brain Items

Table 5 shows the frequency of correct, incorrect and uncertain (‘I don’t know’) answers to GKAB statements, respectively. More than 80% of participants responded correctly to the following six statements: “There are sensitive periods in childhood when it’s easier to learn things.” (93.1%); “Individual learners show preferences for the mode in which they receive information (e.g., visual, auditory, kinesthetic).” (91.7%); “Vigorous exercise can improve mental function.” (83.2%); “Normal development of the human brain involves the birth and death of brain cells.” (83.1%); “Extended rehearsal of some mental processes can change the shape and structure of some parts of the brain.” (82.2%); “Mental capacity is hereditary and cannot be changed by the environment or experience.” (80.2%).

The most frequent incorrect responses were the following: “The left and right hemisphere of the brain always work together.” (49.3%); “Production of new connections in the brain can continue into old age.” (33.6%); “Learning is not due to the addition of new cells to the brain.” (29.4%).

In average, uncertainty was greater compared with that in case of neuromyths. The rate of ‘I don’t know’ answers varied between 1.1% (“Mental capacity is hereditary and cannot be changed by the environment or experience.”) and 26.9% (“Learning is not due to the addition of new cells to the brain.”).

### 3.6. Comparison of Answers to Neuromyths and to Statements Related to General Knowledge about the Brain

**Figure 2:**
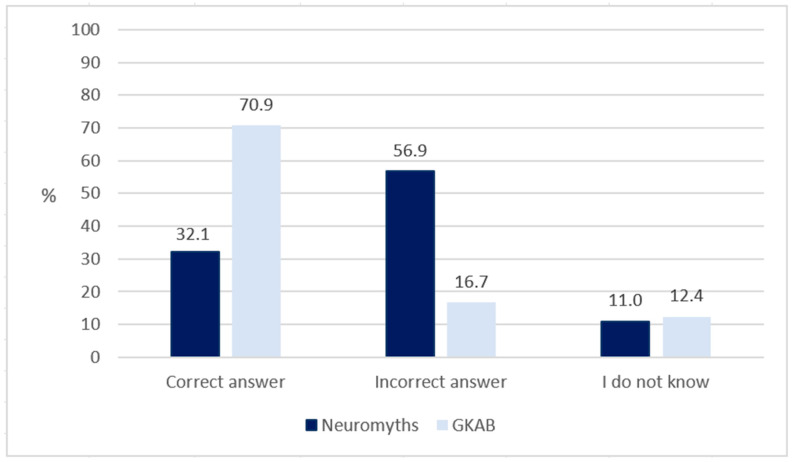
Average rates (%) of correct, incorrect and ‘I do not know’ answers to neuromyth-related and general knowledge about the brain (GKAB) statements.

Figure 2 shows the average rate of correct, incorrect and ‘I don’t know’ answers among participants to NM and GKAB statements, respectively.

In the case of the 10 neuromyths, the average rate of correct answers was 32.10% (range: 3.0–75.1%), the average rate of incorrect answers was 56.93% (range: 16.1–89.7%), and the average rate of uncertain (’I don’t now’) answers was 10.94% (range: 7.2–18.2%).

In the case of the 13 GKAB statements, the average rate of correct answers was 70.92% (range: 33.8–93.1%), the average rate of incorrect answers was 16.67% (range: 2.2–49.3%), and the average rate of uncertain answers was 12.29% (range: 1.1–26.9%).

### 3.7. Comparison with Previous Studies

To date, few studies have assessed the neuroliteracy of pre-service teachers. Table 6 shows the neuromyth error scores (i.e., the rates of incorrect answers to NM statements) and the GKAB scores (i.e., the rates of correct answers to GKAB statements) in the current study and in eight previous studies from other countries that also included pre-service teachers. The Hungarian sample has the second highest endorsement of neuromyths (56.90%, range: 84.46–37.2%). GKAB score is in the middle, ranked as fifth among the eight studies (70.92%, range: 50.1–87.7%).

### 3.8. Factors Associated with the Prevalence of NM and GKAB Scores

The results of ordinal logistic regression analysis are presented in Table S1. Together, the examined factors accounted for a significant amount of variance in the prevalence of NM (likelihood ratio χ^2^ (16) = 38.352, *p* < 0.001). The fitting model accounted for approximately 5% of the variance in the outcomes (Nagelkerke pseudo-R^2^ = 0.049). The model predicting the incorrect answers to GKAB was not significant (likelihood ratio χ^2^ (16) = 25.71, *p* = 0.107).

Men were more likely to believe in NM than women (B = −0.570, SE = 0.243, OR = 0.57, *p* < 0.05), and the frequency of using Facebook to obtain information about neuroscience (B = 0.286, SE = 0.072, OR = 1.33, *p* < 0.001) significantly and independently increased the prevalence of NM.

The rate of incorrect answers to GKAB did not show statistically significant associations with the examined factors, including NM error scores. Interestingly, indicators of previous education (previous degree, number of completed academic years, psychology courses and courses related to neuroeducation) and interest in neuroscience of learning/behavior did not significantly affect the responses.

## 4. Discussion

To our knowledge, this is the first study assessing the prevalence of neuromyths in Hungary, and the first study assessing pre-service teachers’ attitude to neuromyths in Central and Eastern Europe. Our results show that Hungarian pre-service teachers believe in most of the previously studied educational neuromyths.

### 4.1. Endorsement of Neuromyths and General Knowledge about the Brain

In total, 6 neuromyths were considered true by more than half of participants, and the mean error score (i.e., rate of incorrect answers) for all 10 NM statements was 56.9%. This value ranked the second highest in comparison with previous studies from other countries (Table 6), only preceded by an Australian result (Kim and Sankey 2018).

In our study, the most prevalent neuromyths among pre-service teachers were the following: “Exercises that rehearse co-ordination of motor-perception skills can improve literacy skills.” (89.7%); “Children have learning styles that are dominated by particular senses.” (82.1%) and “Short bouts of co-ordination exercises can improve integration of left and right hemispheric brain function.” (81.4%). The least prevalent neuromyth was the following: “Learning problems associated with developmental differences in brain function cannot be remediated by education.” (16.1%), which is consistent with the findings of multiple previous studies (Dündar and Gündüz 2016; Ferrero et al. 2016; Papadatou-Pastou et al. 2017).

Interestingly, the neuromyths related to movement and motor training had the highest acceptance rate in our sample. As mentioned above, the most widely accepted neuromyth among Hungarian pre-service teachers was the false belief in the effect of motor coordination exercises on literacy, and not the myth related to learning styles, which was found to be the most prevalent in many other studies including in-service teachers (Dekker et al. 2012; Grospietsch and Mayer 2019; Janati Idrissi et al. 2020; Karakus et al. 2015) and pre-service teachers (Ching et al. 2020; Dündar and Gündüz 2016; Kim and Sankey 2018). Another widely believed movement-related neuromyth is the presumed effect of coordination exercises on interhemispheric integration.

We posit that the high prevalence of movement-related neuromyths might be partly attributable to the strong presence and great popularity of various movement-based, perceptuomotor, sensorimotor and reflex integration therapies (e.g., Doman-Delecato therapy, INPP therapy, Ayres therapy, Planned Sensorimotor Therapy, Brain Gym) in Hungary. These type of therapies, which are considered to be controversial by many experts (reviewed by Hyatt et al. 2009), have become widespread over the past decade in Hungary and are now available in practically every level of the public education system (Szvatkó et al. 2021). The continuous promotion of such interventions in the mass media and social media, as well as in trainings and conferences available to in-service teachers on these topics also facilitate the dissemination of these misconceptions. We are aware of the fact that many lecturers at teacher training universities have completed trainings in one or more of the above mentioned alternative therapies. If such trainings supported their endorsement of misconceptions, it might affect their students’ (and thus, our study participants’) attitudes to such therapies and misdirect their future teaching practices. Further comparative studies should investigate this hypothesis.

The mean score reflecting general knowledge about the brain (i.e., the average rate of correct answers to the 13 GKAB statements) among Hungarian pre-service teachers was 70.92%, which is relatively high compared with similar studies published in Turkey (Dündar and Gündüz 2016), the USA (Ruhaak and Cook 2018) and Ecuador (Falquez Torres and Ocampo Alvarado 2018). Even higher scores were reported from Greece (Papadatou-Pastou et al. 2017), Chile (Ferreira and Rodríguez 2022) and Australia (Carter et al. 2020).

Of the 13 GKAB statements, the mean score was above 50% in case of 10 statements and below 50% in case of 3 statements. Statements with the highest rates of correct answers were the following: “There are sensitive periods in childhood when it’s easier to learn things.” (93.1%) and “Individual learners show preferences for the mode in which they receive information (e.g., visual, auditory, kinesthetic).” (91.7%). Both values are consistent with other studies, including pre-service teachers (Dündar and Gündüz 2016; Grospietsch and Mayer 2019; Papadatou-Pastou et al. 2017).

Interestingly, the lowest rate of correct answers (33.8%) was found for the following GKAB statement: “The left and right hemisphere of the brain always work together.” This statement is particularly important from an educational perspective. First, many “brain-based” educational programs claim to target the assumed “problems” of lateralization and interhemispheric integration. These approaches are usually based on outdated theories, such as Orton’s mixed cerebral dominance theory (Orton 1937), Delacato’s neurological repatterning theory (Delacato 1959), that have been widely criticized (AAP 2010; Committee on Children with Disabilities 1999; Kroeze et al. 2016). A well-known example is Brain Gym, also known as educational kinesiology (Dennison and Dennison 1994), which is a widely used program in many countries all around the globe—including Hungary—despite the lack of any sound theoretical or empirical evidence (Hyatt 2007) for its validity. In a recent position statement article, Kroeze et al. (2016, p. 78) concluded that “given the limited time children are able to spend in the classroom environment, educators need to implement practices that have been validated by empirical research and not waste valuable time participating in the nuisance of Brain Gym or other pseudoscientific interventions that claim to provide a magical cure for all that ails humanity.”

Finally, we would like to highlight that a relatively low but still considerable proportion (19.8%) of participants were incorrect or uncertain about the effect of biological programming and genetics on mental capacity, that is, they considered such effects superior to environmental influence. This view contradicts the widely accepted scientific consensus that although mental abilities are influenced by a great number of genetic factors, the manifestation of those genetic determinants is heavily influenced by environmental factors and experience (Davidson and McEwen 2012). The deterministic view of brain development and mental capacity is likely to be linked to some teachers’ fixed mindsets and their views on innate intelligence (Dweck 2006; Dweck and Leggett 1988), and might influence teachers’ practices in an undesirable way.

### 4.2. Factors Influencing Neuroliteracy

The results of ordinal logistic regression analysis are presented in Table S1. In the present study, the examined predictors for the incorrect answers to NM and GKAB were the following: gender, age, previous degree, academic year, completed psychological courses, current specialization in university education, previous courses related to neuroeducation, interest in neuroscience of learning/behavior, frequency of using information sources about neuroscience.

The results of our ordinal logistic regression analysis showed that two predictors, male gender and the frequency of using Facebook to obtain information about neuroscience independently increased the prevalence of false beliefs in NM. The statistical model predicting the incorrect answers related to GKAB was not significant, which means that none of the examined predictors were associated with GKAB error score.

Previous studies had inconsistent results regarding the role of gender in the tendency to accept NM. In our study, the frequency of incorrect answers was slightly higher among men than among women, that is, men were more likely to believe in NM than women. This finding is similar to those of Dündar and Gündüz (2016), but different from the findings of Ferrero et al. (2016) who reported a higher rate of incorrect answers among women and to other studies that found no significant difference between men and women (e.g., Hermida et al. 2016; Karakus et al. 2015).

Interestingly, indicators of previous education (previous degree, the number of completed academic year, psychology courses and courses related to neuroeducation) and interest in neuroscience of learning/behavior did not significantly affect answers among Hungarian pre-service teachers. This finding is consistent with that of Gleichgerrcht et al. (2015) who found that neuroscience education was not an effective protective factor against misconceptions. Other studies, however, found a protective effect of education courses against neuromyths (Macdonald et al. 2017; Ruhaak and Cook 2018).

Older age, having a degree, having completed psychological courses, spending more time in teacher training and using various sources of information on neuroscience did not demonstrate any protective effects against NM (i.e., none of these factors decreased the rate of incorrect answers).

The above findings suggest—somewhat concerningly—that during their university studies, neuroscience literacy of pre-service teachers does not develop in a desirable way: it is mostly their misconceptions that are reinforced during their studies. These findings are in contrast with those of a Turkish study, in which neuromyths scores declined during the first 3 years of training, but increased again in the fourth year, which the authors explained with the involvement of neuroscience in senior-grade teachers’ practical courses (Dündar and Gündüz 2016).

Besides university courses, the participants reported to mostly obtain neuroscience-related information from non-scientific sources, such as YouTube, magazines and Facebook. More reliable sources, such as scientific papers and conferences are used by only a small subgroup of participants. We find these preferences problematic as the frequency of using Facebook as the main source of neuroscientific information was associated with a higher rate of incorrect answers. Thus, pre-service teachers prefer sources of neuroscience-related information that actually strengthen their misbeliefs.

In our study, better GKAB was not a protective factor against NM. Previous studies were inconsistent in this respect, although our statistical analysis (multivariate ordinal logistic regression) was more robust in comparison with correlational analysis. Some correlational studies found positive correlations (e.g., Canbulat and Kiriktas 2017; Kim and Sankey 2018; Papadatou-Pastou et al. 2017; van Dijk and Lane 2020), whereas others found no or an inverse correlation (Dekker et al. 2012; Zhang et al. 2019).

## 5. Limitations

Our results are limited in their interpretation by a certain number of factors. First, our questionnaire was adapted from English into Hungarian. In the adaptation process, some items of the original Dekker et al. (2012) questionnaire were excluded. Our expert decisions during the adaptation process were based on several factors. Some statements were culturally inadequate in the education field in Hungary (e.g., “Regular drinking of caffeinated drinks reduces alertness”). Other statements were excluded on the basis of concerns regarding the evidence supporting them (Sullivan et al. 2021), such as “Circadian rhythms (“body-clock”) shift during adolescence, causing pupils to be tired during the first lessons of the school day.” and “It has been scientifically proven that fatty acid supplements (omega-3 and omega-6) have a positive effect on academic achievement”.

Moreover, the questionnaires were formulated so that participants could provide answers on a Likert-scale of agreement, disagreement or uncertainty, which is different from the true/false/’I don’t know’ scoring used by several other studies, including the original Dekker et al. (2012) questionnaire. This transformation might limit the comparability of our results with previously published data.

Since the questionnaire was conducted online, we cannot rule out the possibility that some participants used some research or asked each other before answering the questions, so the answers might not always reflect their own knowledge. Furthermore, self-reported beliefs and answers are known to be influenced by their perceived social desirability, which might override the participants’ own opinions.

## 6. Conclusions

On the basis of our data, we believe that current teacher training in Hungary is not efficient in providing up-to-date information about neuroscience and safeguarding students against neuromyths. Furthermore, Hungarian pre-service teachers seem to choose and/or to have access to inadequate extracurricular sources of neuroscience-related information. Because of these factors, pre-service teachers are not provided with the knowledge that would be necessary for a critical evaluation of nonscientific, profit-oriented programs and their misleading statements, which are heavily marketed and widely available in Hungary. Further research is needed to explore the effects of widely used and promoted perceptuomotor, sensorimotor and reflex therapies on neuromyths. We believe that the above-mentioned problems should be addressed by including evidence-based neuroscience courses in teacher training, in which neuromyths and the nature of scientific misconceptions in general are discussed, the significance of using evidence-based approaches is highlighted and critical thinking is encouraged.

## Figures and Tables

**Table 1:** Coding scheme of the 23 neuroliteracy statements.

	Responder Agrees (Strongly or Somewhat Agrees)	Responder Disagrees (Strongly or Somewhat Disagrees)	Responder Does Not Know
**True statement**	Correct	Incorrect	Uncertain
**False statement**	Incorrect	Correct	Uncertain

**Table 2:** Participants’ demographic characteristics.

Predictor Variable		n	%
Gender	Male	96	11.7
	Female	721	87.7
	Not specified	5	0.6
Previous education	University degree	285	34.7
	No degree	537	65.3
Academic year	1st year	236	28.7
	2nd year	173	21.0
	3rd year	262	31.9
	4th year	88	10.7
	5th year	37	4.5
	6th year	26	3.2
Residence in country regions	Budapest (capital)	178	21.7
	Southern Great plain	42	5.1
	Southern Transdanubia	61	7.4
	Northern Great Plain	95	11.6
	Northern Hungary	87	10.6
	Central Transdanubia	117	14.2
	Central Hungary	183	22.3
	Western Transdanubia	59	7.2
Current specialization in university education	Preschool teacher	144	17.5
	Primary school teacher	118	14.4
	Special needs education teacher	333	40.5
	Physical education teacher	112	13.6
	Other specialized teacher	115	14.0

**Table 3:** Previous education and interest in neuroscience.

Predictor Variable		n	%
Previous degree	In education	149	18.1
	Not in education	136	16.5
	No previous degree	537	65.3
Completed psychology courses	None	35	4.3
	1 course	178	21.7
	2 courses	74	9.0
	3 courses	535	65.1
Previous courses related to neuroeducation	None	596	72.5
	1 course	74	9.0
	Multiple courses	33	4.0
	Not sure	119	14.5
Interest in neuroscience of learning/behavior	Not at all	20	2.4
	Not really	45	5.5
	To some extent	235	28.6
	Definitely	303	36.9
	Very much	219	26.6

**Table 4:** Participants’ answers to statements related to educational neuromyths (in descending order of the rate of incorrect answers).

Statement ^1^	Participants’ Answers (%)		
	Correct	Incorrect	Uncertain
Exercises that rehearse co-ordination of motor-perception skills can improve literacy skills. (F)	3.0	89.7	7.3
Children have learning styles that are dominated by particular senses. (F)	7.0	82.1	10.9
Short bouts of co-ordination exercises can improve integration of left and right hemispheric brain function. (F)	3.5	81.4	15.1
Children are less attentive after consuming sugary drinks and/or snacks. (F)	27.7	62.4	9.9
Differences in hemispheric dominance (left brain, right brain) can help explain individual differences amongst learners. (F)	24.8	60.0	15.2
There are critical periods in childhood after which certain things can no longer be learned. (F)	32.6	59.9	7.5
We only use 10% of our brain. (F)	33.8	48.0	18.2
Children must acquire their native language before a second language is learned. If they do not do so neither language will be fully acquired. (F)	57.3	35.5	7.2
The left and right hemisphere of the brain work independently. (F)	56.1	34.2	9.7
Learning problems associated with developmental differences in brain function cannot be remediated by education. (F)	75.5	16.1	8.4

^1^ Note. All statements are false on the basis of the available scientific evidence, following Dekker et al. (2012).

**Table 5:** Participants’ answers to statements related to general knowledge about the brain (in descending order of the rate of correct answers).

Statement (True/False) ^1^	Participants’ Answers (%)		
	Correct	Incorrect	Uncertain
There are sensitive periods in childhood when it’s easier to learn things. (T)	93.1	2.3	4.6
Individual learners show preferences for the mode in which they receive information (e.g., visual, auditory, kinesthetic). (T)	91.7	2.2	6.1
Vigorous exercise can improve mental function. (T)	83.2	8.3	8.5
Normal development of the human brain involves the birth and death of brain cells. (T)	83.1	6.9	10.0
Extended rehearsal of some mental processes can change the shape and structure of some parts of the brain. (T)	82.2	7.3	10.5
Mental capacity is hereditary and cannot be changed by the environment or experience. (F)	80.2	18.7	1.1
Learning occurs through modification of the brains’ neural connections. (T)	72.6	5.4	22.0
Brain development has finished by the time children reach secondary school. (F)	71.7	14.3	14.0
We use our brains 24 h a day. (T)	71.3	22.4	6.3
When a brain region is damaged other parts of the brain can take up its function. (T)	70.0	16.6	13.4
Production of new connections in the brain can continue into old age. (T)	45.4	33.6	21.0
Learning is not due to the addition of new cells to the brain. (T)	43.7	29.4	26.9
The left and right hemisphere of the brain always work together. (T)	33.8	49.3	16.9

^1^ Note. Statements are labeled true (T) or false (F) on the basis of the available scientific evidence, following Dekker et al. (2012).

**Table 6:** Prevalence of neuromyths (i.e., rates of incorrect answers to NM statements) and GKAB scores (i.e., rates of correct answers to GKAB statements) across studies.

Study (Year)	Country	Prevalence of NM (%)	Score in GKAB (%)
1. Kim and Sankey (2018) ^1^	Australia	84.46	75.60
**2. Current study**	**Hungary**	**56.90**	**70.92**
3. Ferreira and Rodríguez (2022)	Chile	56.70	77.50
4. Falquez Torres and Ocampo Alvarado (2018)	Ecuador	56	54
5. Škraban et al. (2018)	Slovenia	53.86	not studied
6. Dündar and Gündüz (2016)	Turkey	52.72	50.10
7. Ruhaak and Cook (2018)	USA	51.24	62.50
8. Papadatou-Pastou et al. (2017)	Greece	43.62	78.94
9. Carter et al. (2020)	Australia	37.2	87.7

^1^ Note: Only 5 neuromyth statements were included in the questionnaire.

## Data Availability

Data are available upon request.

## Supplementary Materials

The following supporting information can be downloaded at: https://www.mdpi.com/article/10.3390/jintelligence11020031/s1, Table S1: Results of ordinal logistic regression representing odds ratios (OR) with 95% confidence interval.
