# Supplementary material for: The Prevalence of Educational Neuromyths among Hungarian Pre-Service Teachers
Source: J Intell. 2023 Feb 3;11(2):31. doi: 10.3390/jintelligence11020031 (PMC9963927; doi:10.3390/jintelligence11020031)
Supplement: Supplementary file 1 [file jintelligence-11-00031-s001.zip › jintelligence-2101979-supplementary.pdf]

The Prevalence of Educational Neuromyths among Hungarian Pre-service Teachers

Supplementary materials

Table S1: Results of ordinal logistic regression representing odds ratios (OR) with 95% confidence interval

Factors		Rate of NM[#] incorrect answers
**Gender**		**0.57 (0.35-0.91)**
**Age**		1.01 (1.00-1.03)
**Previous education**	**Previous degree**	0.93 (0.62-1.39)
	**Academic year**	1.04 (0.91-1.20)
	**Number of completed psychology courses**	1.06 (0.89-1.27)
	**Current specialization**	0.95 (0.86-1.05)
	**Previous courses related to neuroeducation**	0.99 (0.74-1.31)
**Interest in neuroscience of learning/behavior**		0.96 (0.82-1.12)
**GKAB* error score**		1.01.(0.99-1.02)
**Frequency of using information sources**	**Facebook**	**1.33 (1.16-1.53)**
	**YouTube**	0.96 (0.84-1.11)
	**Magazine**	0.98 (0.84-1.15)
	**Journal**	1.01 (0.86-1.19)
	**Conference**	0.99 (0.79-1.24)
	**Training**	1.02 (0.83-1.26)
	**Science journal**	1.13 (0.93-1.36)

Note: Significant ORs are marked with **bold letters** (p<0.001); *GKAB=General Knowledge About the Brain; [#]NM=Neuromyth **0.57 (0.35-0.91)**
